# Association between Dietary Magnesium Intake and Glycemic Markers in Ghanaian Women of Reproductive Age: A Pilot Cross-Sectional Study

**DOI:** 10.3390/nu13114141

**Published:** 2021-11-19

**Authors:** Helena J. Bentil, Alyssa M. Abreu, Seth Adu-Afarwuah, Joseph S. Rossi, Alison Tovar, Brietta M. Oaks

**Affiliations:** 1 Department of Nutrition and Food Sciences, University of Rhode Island, Kingston, RI 02881, USA; amabreu@uri.edu (A.M.A.); alison_tovar@uri.edu (A.T.); boaks@uri.edu (B.M.O.); 2 Department of Nutrition and Food Science, University of Ghana, Accra LG 25, Ghana; sadu-afarwuah@ug.edu.gh; 3 Department of Psychology, University of Rhode Island, Kingston, RI 02881, USA; jsrossi@uri.edu

**Keywords:** magnesium, diabetes, nutrition transition, women of reproductive age, Ghana

## Abstract

Low magnesium intake has been shown to be associated with an increased risk of type 2 diabetes mellitus (T2DM) in several studies conducted in high-income countries. However, very few studies have been performed in Africa, where many countries have a growing rate of T2DM. We conducted a pilot cross-sectional study among 63 women in Ghana to investigate the association between magnesium intake and glycemic markers. We assessed dietary magnesium using a food frequency questionnaire and glycemic markers using fasting blood glucose and glycated hemoglobin A1c (HbA1c). Our findings showed that the mean magnesium intake was 200 ± 116 mg/day. The prevalence of T2DM was 5% by measuring fasting blood glucose and 8% by measuring HbA1c. Unadjusted linear regression models revealed that higher magnesium intake significantly predicted higher fasting blood glucose levels (β = 0.31; 95% CI: 0.07, 0.55; *p* = 0.01) and HbA1c levels (β = 0.26; 95% CI: 0.01, 0.51; *p* = 0.04). In adjusted analyses, magnesium intake was no longer significantly associated with either fasting blood glucose levels (β = 0.22; 95% CI: −0.03, 0.46; *p* = 0.08) or HbA1c levels (β = 0.15; 95% CI: −0.08, 0.39; *p* = 0.20). In conclusion, our study did not show a significant association between magnesium intake and glycemic markers in women of reproductive age in Ghana. The results of this study need to be further substantiated because this was the first study to examine magnesium intake and glycemic markers in this population in Africa.

## 1. Introduction

Magnesium is a critical mineral in the body serving as a cofactor for more than 300 enzymes that regulate diverse biochemical reactions, including blood glucose [1]. Magnesium deficiency can affect insulin regulation and may increase the risk of diabetes due to its essential role in the activation of the tyrosine kinase enzyme for insulin receptor activity [2]. Thus, magnesium deficiency could impair the insulin signal transduction pathway by interfering with the tyrosine kinase activity of the insulin receptor [1,2].

Several studies have reported that low magnesium intake is associated with type 2 diabetes mellitus (T2DM) [3,4,5,6], and randomized controlled trials show that magnesium supplementation improves glucose parameters in adults with diabetes and improves insulin sensitivity in those at high risk of diabetes [7,8,9,10]. However, the majority of research has been conducted in high-income countries [3,4,5,6,7,8,9,10], and no study has examined the relationship between magnesium intake and T2DM in Africa.

Ghana, like many countries in Africa, is experiencing a nutrition transition, with less intake of traditional foods and increased consumption of processed foods typically associated with a western diet [11]. As magnesium is present in many traditional Ghanaian foods [12] and typically absent in processed foods [13], magnesium intake has likely been decreasing in Ghana. Simultaneously, the rate of T2DM has been increasing and it is now estimated that among women (aged 18 years and over) in Ghana, T2DM has increased from 5.0% in 2000 to 6.6% in 2014 [14]. The increase in T2DM is particularly concerning for women of reproductive age, as diabetes during pregnancy can lead to adverse birth outcomes such as restricted fetal growth, intrauterine growth restriction, and preterm labor, as well as an increased risk of future diabetes [15,16,17]. Furthermore, low magnesium consumption during pregnancy plays a role in the fetal programming of adult disease [15]. Intrauterine magnesium deficiency in the fetus was linked to insulin resistance after birth, increasing the risk of metabolic syndrome in adulthood [15]. It is unknown whether magnesium deficiency is a factor contributing to the prevalence of T2DM in Ghana as the average magnesium intake in the current diet is unknown. Therefore, this study intends to fill a critical void in our understanding of diet and T2DM risk in Ghana.

To address this knowledge gap, we conducted a pilot study to investigate the association between dietary magnesium intake and two glycemic markers of T2DM risk: fasting blood glucose and glycated hemoglobin A1c (HbA1c). We hypothesized that lower dietary magnesium intake would be associated with higher fasting blood glucose and higher HbA1c.

## 2. Materials and Methods

### 2.1. Study Design, Setting, and Participants

We conducted a pilot cross-sectional study between July and August 2019 among women of reproductive age living in Odumase Krobo, a peri-urban area and the district capital of Lower Manya Krobo District in the Eastern Region of Ghana, about 70 km north of the national capital, Accra. The study was approved by the Ghana Health Service Ethical IRB (REF# GHS-ERC016/10/18) and the University of Rhode Island IRB (REF# BI1819-005). We obtained written informed consent from all study participants.

To be eligible for enrollment, women had to: (a) be between 18 and 49 years old; (b) reside in Odumase Krobo; (c) be able to speak Krobo, Twi, or English; (d) and be non-pregnant by self-report. Pregnant women were excluded because their blood glucose fluctuates during pregnancy. We decided on a sample size of 60 based on a recommendation by Sim et al. [18] for pilot studies.

A list of census enumeration areas for Odumase Krobo was obtained from the Ghana Statistical Services. The list had a total of 19 enumeration areas (EAs) in ascending order according to the EAs ID code/number. Out of the total 19 EAs, 5 EAs were randomly selected. To select the 5 EAs, we first calculated the interval rate by dividing the total number of EAs (19) by the number of EAs to be selected (5), which is approximately 4. Thus, for the 5 EAs, we selected every fourth EA from the list of total EAs. These included the fourth, eighth, twelfth, sixteenth, and eighteenth enumeration areas. We selected a total of 12 households per EA. Every second household that had women within the ages of 18–49 years was selected from the list of households in each EA.

The selected households were visited, and women in each household were further screened for eligibility. Women who were interested and eligible to participate gave their informed consent by either signature or thumbprint. If the eligible woman in the selected household was not interested, the next household was selected from the list of households until a sample size of 60 was obtained. In situations where there was more than one eligible woman in a household, only one was selected using the simple random sampling procedure (balloting technique). Ballot papers equivalent to the number of eligible women in the household were all labeled “No” except for one, which was labeled “Yes”, and were folded and presented to the women for them to choose. The eligible woman who picked the paper labeled “Yes” was enrolled in the study.

All enrolled women received two home visits within two days. During the first home visit, which took approximately two hours, women consented and completed an interviewer-administered survey and a food frequency questionnaire (FFQ) to collect information on socio-demographic characteristics and dietary magnesium intake, respectively as well as height and weight measurements. Blood tests were performed during the second home visit, which took about a half-hour, to collect information on hemoglobin concentrations, fasting blood glucose, and HbA1c.

### 2.2. Study Data Collection

An interviewer-administered questionnaire was used to collect data on socio-demographic characteristics, including age, ethnicity, marital status, number of children, educational level, occupation, and living conditions, including sources of drinking water, sanitation facility, type of building material, type of flooring, and household ownership of assets (e.g., electricity, car, refrigerator/freezer, television, video player, computer, gas/electric stove, and air conditioner). We assessed household socioeconomic status from the reported household’s ownership of the following 18 items: color/black TV, fixed telephone, mobile phone, generator, computer/laptop, refrigerator, freezer, washing machine, sewing machine, radio, bed, DVD/VCD, bicycle, motorcycle, table, cabinet/cupboard, camera, and car/truck, as well as access to internet and electricity. Based on these parameters, the wealth index was determined using principal components analysis and categorized into quintiles (lowest, second, middle, fourth, and highest).

To estimate body mass index (BMI), we measured height and weight using standard procedures with a height board (UNICEF S0114540) and a digital weight scale (SECA 874, Chino, CA, USA), respectively. Body mass index (BMI) was calculated as weight (kg)/height (m)^2^. Study participants were classified as underweight (BMI < 18.5 kg/m^2^), normal weight (BMI 18.5–24.9 kg/m^2^), overweight (BMI 25.0–30.0 kg/m^2^), or obese (BMI ≥ 30.0 kg/m^2^), which was assessed according to the cut-off values by the World Health Organization [19].

We collected information on dietary magnesium intake using a semi-quantitative 150-item food frequency questionnaire (FFQ) based on the FFQ used in larger studies and national surveys in Ghana [20,21,22]. For each food item, we assigned a portion size using standard household measures such as cups, tablespoons, teaspoons, or glasses as well as using photographs from the Food Amounts Booklet [23]. Study participants were asked how often, on average, they consumed the specified amount in the past 7 days. The frequency of consumption of specified portion size was asked in six categories: never, once per week, 2 times per week, 3–4 times per week, 5–6 times per week, once per day, and 2 or more times per day. We calculated magnesium intake by multiplying the frequency of consumption of each food item by the magnesium content of the specified portion size using ESHA Food Processor Nutrition Analysis software, version 10.8 (ESHA Research Inc, 2010, Salem, OR, USA) and the West African Food Composition table. We also collected information on dietary supplement intake. However, none of the reported dietary supplements contained magnesium and were therefore not included in the analysis.

We obtained blood samples from the study participants after an overnight fast following a finger prick with disposable lancets on the second home visit. The markers of glycemia assessed were fasting blood glucose and glycated hemoglobin A1c (HbA1c). We measured fasting blood glucose (FBG) and HbA1c levels once using a glucose meter (AccuChek Aviva Plus) and an HbA1c meter (A1CNow+ System PTS Diagnostics, Whitestown, IN, USA), respectively. Women at risk of diabetes were defined as having an FBG ≥ 126 mg/dL or HbA1C ≥ 6.5%, women at risk of prediabetes were defined as having an FBG of 100–125 mg/dL or HbA1C of 5.7–6.4%, and normal glycemia as having an FBG less than 100 mg/dL or HbA1c less than 5.7% according to the International Diabetes Federation American Diabetes Association criteria [24]. We also measured hemoglobin concentrations. A drop of blood was drawn into a microcuvette and analyzed using a HemoCue™ Hb 201 Analyzer (Radiometer Medical Aps, Brønshøj, Denmark). Study participants were classified as normal (Hb ≥ 12.0 g/dL), mild anemia (Hb 11.0–11.9 g /dL), moderate anemia (Hb 8.0–10.9 g/dL), or severe anemia (Hb < 8.0 g/dL), and were assessed according to the cut-off values by the World Health Organization [25].

### 2.3. Statistical Analysis

We analyzed the study data with SAS Studio 5.2 (SAS Institute, Cary, NC, USA). Continuous variables are presented as mean ± SD, and categorical variables as frequency (%). Multiple linear regression was used to determine the association between dietary magnesium intake and blood glucose concentration. The dependent variables were the glycemic markers (fasting blood glucose and HbA1c), and dietary magnesium intake was the independent variable. We used the Shapiro–Wilk test to check the normality of residuals and inversely transformed the outcome variable, fasting blood glucose. For the adjusted regression analysis, bivariate analysis was first performed to determine whether socio-demographic variables, BMI, and hemoglobin concentration, which were selected based on prior literature [26,27,28], were significantly associated with both the dependent and independent variables. Variables found to be statistically significant at *p* < 0.10 were included as covariates in the adjusted regression models. Age was the only covariate found to be significantly associated with both the dependent variables (fasting blood glucose or HbA1c) and the independent variable (magnesium intake). BMI was also included as a covariate because of its association with blood glucose concentrations [28]. The independent variable and covariates in the adjusted model were checked for multicollinearity by estimating the variance inflation factor (VIF) for all variables; no correlation was identified between the variables (VIF < 2). Upon examining studentized residuals larger than 3 standard deviations, we determined that there was an outlier in magnesium intake. Given that the intake was still considered plausible (658 mg/day) [29], we decided to keep this value in the regression model. However, we conducted a post hoc linear regression where we split magnesium intake into 3 groups to test the relationship between magnesium intake and the glycemic markers and thus mitigated the effect of the outlier in the magnesium intake distribution. We also performed a post hoc analysis of logistic regression and predicted the risk of hyperglycemia (FBG ≥ 100 mg/dL or HbA1c ≥ 5.7%) according to magnesium intake. A *p*-value of <0.05 was considered statistically significant. Statistical power analysis was conducted for a multiple regression analysis consisting of four potential covariates and assuming alpha = 0.05 for a planned sample size of 60 study participants. The results suggested that power would be 0.80 for an overall model R^2^ of 0.18 and that a model R^2^ of 0.10 or greater would be detected as statistically significant at *p* < 0.05.

## 3. Results

### 3.1. Background Characteristics of Study Participants

Table 1 summarizes the background characteristics of the study participants. A total of 63 women were enrolled in the study. The mean ± SD age of the women was 29.5 ± 8.5 years. The majority of the women were Ga-Adangmes (98.4%), had attended school (88.9%), were employed (61.9%), and had one or more children (77.8%). About 30% were classified as overweight, 14% were classified as obese, and only 1.6% were classified as underweight. More than half of the women were anemic (57.1%), and 3% of the women were severely anemic. Most of the women in our study had normal glycemia: fasting blood glucose (55.6%) vs. HbA1c (67.2%). About 40% and 25% of the women were classified as having prediabetes per fasting blood glucose and HbA1c levels, respectively, while 5% (from fasting blood glucose measurements) vs. 8% (from HbA1c measurements) were classified as having diabetes. The mean ± SD daily intake of magnesium in the study population was 200 ± 116 mg. The majority of the women (84%) in this study did not meet the recommended daily magnesium intake as required by their age. The daily recommendation by age is 360 mg for 14–18 years, 310 mg for 19–30 years, and 320 mg for 31+ years.

### 3.2. Association between Magnesium Intake and Glycemic Markers 

Table 2 shows the associations between magnesium intake and glycemic markers. In both unadjusted models, higher magnesium intake was significantly associated with higher fasting blood glucose levels (β = 0.31, *p* = 0.01) and higher HbA1c levels (β = 0.26, *p* = 0.04). This means that a one SD increase in magnesium intake (1 SD = 116 mg/d) was associated with a higher fasting blood glucose of 0.31 mg/dL (95% CI: 0.07, 0.55) and a higher HbA1c of 0.26% (95% CI: 0.01, 0.51). After adjusting for covariates, magnesium intake was no longer significantly associated with fasting blood glucose (β = 0.22, 95% CI: −0.03, 0.46, *p* = 0.08) and HbA1c levels (β = 0.15, 95% CI: −0.08, 0.39, *p* = 0.20). In our post hoc tertile analysis, presented in Supplementary Table S1, the adjusted models also showed no relationship between magnesium intake and fasting blood glucose (β = 0.10, 95% CI: −0.15, 0.35, *p* = 0.43) and HbA1c (β = 0.18, 95% CI: −0.04, 0.32, *p* = 0.13). However, the unadjusted models, showed a non-significant relationship between magnesium intake and fasting blood glucose (β = 0.20, 95% CI: −0.05, 0.45, *p* = 0.12) and HbA1c levels (β = 0.26, 95% CI: −0.004, 0.39, *p* = 0.05).

### 3.3. Post Hoc Analysis Showing Risk of Hyperglycemia in Women of Reproductive Age According to Magnesium Intake

Logistic regression was performed to predict hyperglycemia based on magnesium intake categorized according to the RDA by age for magnesium (Table 3). Both unadjusted and adjusted models showed no significant relationship between magnesium intake and glycemic markers.

## 4. Discussion

The aim of this study was to explore the association between magnesium intake and risk of type 2 diabetes in a sample of women of reproductive age in Ghana. Our study showed no associations between magnesium intake and glycemic markers (fasting blood glucose and HbA1c) after adjusting for covariates. This, however, contrasts the findings of other studies. A recent meta-analysis of 41 prospective cohort studies found a significant inverse association between magnesium intake and risk of type 2 diabetes [5]. Similarly, the Black Women’s Health Study, an 8-year prospective study of 41,186 U.S. Black women found that a higher dietary magnesium intake was associated with a lower risk of type 2 diabetes [30]. This contrast could be due to differences in geographic regions as several of these studies were conducted in Europe, Australia, North America, and Asia. There are no previously published studies conducted in African populations; therefore, further research, especially longitudinal studies, is warranted to confirm our results. There are some studies, however, that have found no association between magnesium intake and risk of type 2 diabetes [31,32]. In particular, a double-blind randomized cross-over trial [32], which investigated the effects of magnesium supplementation on metabolic control and insulin sensitivity in 98 type 2 diabetic patients, did not find magnesium supplementation to have any significant improvement in the fasting glucose, HbA1c, insulin, or homeostatic model assessment—insulin resistance (HOMA-IR) of the patients. However, as the study was completed in type 2 diabetic patients, the authors attributed the finding to magnesium loss due to increased urinary magnesium excretion.

Of note, in our unadjusted results, we found positive associations between magnesium intake and glycemic markers, suggesting that a higher magnesium intake predicts higher fasting blood glucose and HbA1c levels in women of reproductive age in Ghana. However, this finding is in contrast to the inverse associations reported in previous studies [3,4,5,7,8,30]. A possible explanation regarding the positive relationship observed between magnesium intake and glycemic markers might be related to the number of factors that interfere with magnesium absorption following intake. Specifically, dietary aluminum has been shown to cause approximately a five-fold reduction in the absorption of magnesium in the gut [13,33]. Apart from the dietary sources of aluminum, the use of aluminum utensils in cooking is considered to result in significant increases in the aluminum content of food due to the leaching of aluminum from aluminum utensils [33,34]. In our study setting, as in many parts of Africa [35], aluminum utensils were used in cooking, which may have contributed to an increase in the intake of this metal in addition to the dietary sources, including vegetables, food additives, cereals, and cereal products [34]. In addition, the major sources of magnesium in our study were millet and maize. These foods, especially millet, are consumed in the form of porridge, hausa koko, which is sweetened with sugar. Thus, although millet porridge is a significant source of magnesium, it also contains sugar, the high intake of which decreases the absorption of magnesium in the gut and causes increased magnesium excretion via the kidneys [13,33]. We acknowledge that though our unadjusted findings may be of note, only showing the bivariate relationship between magnesium intake and risk of type 2 diabetes without controlling for covariates. That said, in our study, adjustment for age and BMI attenuated the effect of our results. We observed higher blood glucose concentrations in older and overweight women than in younger and normal-weight women.

Our study has some limitations. First, the cross-sectional nature of this study means that no causal associations between magnesium intake and risk of type 2 diabetes can be established. Second, we acknowledge that the possible recall bias in the dietary assessment and the common habit for people in Ghana to share or consume food from a shared bowl could have led to underreporting of food intakes. Participants may have had difficulties remembering the foods and quantities they consumed the previous week, which could have affected the reported intake. Third, we had a small sample size and our target population was recruited from a particular region in Ghana, which limits generalizability. However, the diets of the people in this area are roughly similar to those of most people in Ghana. Fourth, magnesium intake from drinking water was not assessed, which is consistent with previous studies [5,6,7,8,9]. The primary source of drinking water in our study was tap water. Tap water in Ghana is softened, which essentially reduces the concentrations of minerals such as magnesium from the water. The mean magnesium concentrations of drinking water vary between 1 and 20 mg/L [36]. Assuming study participants consumed 1 to 1.5 L of water per day, this would correspond to approximately 1 to 30 mg/d of magnesium, and we think that this would not change the null association we found in our study.

Our study has several strengths. We used the random sampling method to recruit participants, reducing bias. We had information about several covariates, including socio-demographic factors, hemoglobin concentrations and BMI for which we were able to perform an adjusted analysis. Additionally, we used two glycemic markers to assess the risk of type 2 diabetes. Fasting blood glucose measures blood glucose level at a single point in time and HbA1c measures blood glucose levels in the previous 3 months, integrating both fasting and postprandial hyperglycemia [37].

In summary, we observed no significant association between magnesium intake and glycemic markers in women of reproductive age in Ghana. Given the role magnesium plays in glucose and insulin homeostasis and the fact that, in this study population, a high percentage of women did not meet the RDA for magnesium, more studies with longitudinal design are needed to confirm the reported results.

## Figures and Tables

**Table 1:** Background characteristics of the study population.

Variable	Frequency (%)/Mean ± SD
Age, years	29.5 ± 8.5
Marital status	
Currently married	28 (44.4%)
Single/separated/widow	35 (55.6%)
Ethnicity	
Ga-Dangme	62 (98.4%)
Others	1 (1.6%)
School Attendance	
Yes	56 (88.9%)
No	7 (11.1%)
Education	
None	7 (11.1%)
KG/primary/JHS (Low)	42 (66.7%)
SHS/tertiary (High)	14 (22.2%)
Occupation	
Not employed	24 (38.1%)
Employed	39 (61.9%)
Number of children	
None	14 (22.2%)
1	13 (20.6%)
≥2	36 (57.1%)
Mean BMI, kg/m^2^	25.2 ± 5.1
BMI Category *n* (%)	
Underweight	1 (1.6%)
Normal	34 (54.0%)
Overweight	19 (30.2%)
Obese	9 (14.3%)
Mean fasting blood glucose, mg/dL	101.2 ± 15.2
Fasting blood glucose category	
Normal (<100 mg/dL)	35 (55.6%)
Impaired fasting glucose (100–126 mg/dL), at risk	25 (39.7%)
Raised fasting blood glucose (≥126 mg/dL), at risk	3 (4.8%)
Mean HbA1c % (*n* = 61)	5.5 ± 0.6
HbA1c category (*n* = 61)	
Normal (<5.7)	41 (67.2%)
Pre-diabetes (5.7–6.4), at risk	15 (24.6%)
Diabetes (≥6.5), at risk	5 (8.2%)
Mean hemoglobin concentration, g/dL	11.5 ±1.7
Anemia *n* (%)	
Normal (≥12 g/dL)	27 (42.9%)
Mild (11.9–11 g/dL)	14 (22.2%)
Moderate (10.9–8 g/dL)	20 (31.8%)
Severe (<8 g/dL)	2 (3.2%)
Mean Magnesium Intake, mg/day	200 ± 116
RDA for Magnesium intake	
Met RDA	10 (15.9%)
Did not meet RDA	53 (84.1%)

**Table 2:** Association between magnesium intake and glycemic markers.

	Fasting Blood Glucose (mg/dL) *n* = 63			HbA1c (%) *n* = 61		
	β	95% CI	*p*-Value	β	95% CI	*p*-Value
Magnesium intake						
Unadjusted	0.31	0.07, 0.55	0.01	0.26	0.01, 0.51	0.04
Adjusted ^1^	0.22	−0.03, 0.46	0.08	0.15	−0.08, 0.39	0.20

^1^ Adjusted for age and BMI.

**Table 3:** Risk of hyperglycemia in women of reproductive age according to magnesium intake.

Magnesium Intake (mg/Day)	Fasting Blood Glucose (mg/dL) *n* = 63						HbA1c (%) *n* = 61					
	Unadjusted			Adjusted ^1^			Unadjusted			Adjusted ^1^		
	OR	95% CI	*p* Value	OR	95% CI	*p*-Value	OR	95% CI	*p* Value	OR	95% CI	*p*-Value
Met RDA	1.00			1.00			1.00			1.00		
Unmet RDA	0.78	0.20, 3.00	0.70	0.89	0.21, 3.72	0.86	0.46	0.12, 1.81	0.26	0.42	0.07, 2.45	0.33

^1^ Adjusted for age and BMI.

## Data Availability

Data is not publicly available, but requests may be sent to the corresponding author, Helena Bentil (helena_bentil@uri.edu) or the principal investigator of the project, Brietta Oaks (boaks@uri.edu).

## Supplementary Materials

The following are available online at https://www.mdpi.com/article/10.3390/nu13114141/s1, Table S1: Association of tertile analysis of magnesium intake and glycemic markers.
