# Supplementary material for: Association between Dietary Magnesium Intake and Glycemic Markers in Ghanaian Women of Reproductive Age: A Pilot Cross-Sectional Study
Source: Nutrients. 2021 Nov 19;13(11):4141. doi: 10.3390/nu13114141 (PMC8619971; doi:10.3390/nu13114141)
Supplement: Supplementary file 1 [file nutrients-13-04141-s001.zip › nutrients-1414800-SI.pdf]

Supplementary Table

Table S1. Association of tertile analysis of magnesium intake and glycemic markers.

	Fasting blood glucose (mg/dL) n=63			HbA1c (%) n=61		
	β	95% CI	P value	β	95% CI	P value
Mg intake[1]						
Unadjusted	0.20	-0.05, 0.45	0.12	0.26	-0.004, 0.39	0.05
Adjusted[2]	0.10	-0.15, 0.35	0.43	0.18	-0.04, 0.32	0.13

[1] Magnesium (Mg) intake categorized into tertiles (42.64mg-129.90mg, 144.36mg-225.64mg and 227.93mg-658.70mg)
[2]Adjusted for age and BMI
